# Cancer-related health behaviors during the COVID 19 pandemic in geographically diverse samples across the US

**DOI:** 10.1186/s12885-024-13373-5

**Published:** 2025-01-09

**Authors:** Breanna B. Greteman, Allison Cole, Mary E. Charlton, Jackilen Shannon, Deanna Kepka, Electra D. Paskett, Evelinn A. Borrayo, Jamie L. Studts, Hayley S. Thompson, Isabel Scarinci, Lynn Chollet Hinton, Elizabeth A. Chrischilles, Crystal J. Garcia-Auguste, Kaila Christini, Heather Aker, Jesse J. Plascak, Felicity W. K. Harper, Monica L. Baskin, Sejong Bae, Vishruti Pandya, Young-il Kim, Babalola Faseru, Christie Befort, Hanluen Kuo, Mark Dignan, Juan Canedo, Victoria Champion, Bettina F. Drake, Kia L. Davis, Debra L. Friedman, Mohamed I. Elsaid, Scherezade K. Mama, Wendy F. Cohn

**Affiliations:** 1 University of Iowa College of Public Health, Holden Comprehensive Cancer Center, Iowa City, IA 52242 USA; 2 University of Washington School of Medicine, Seattle, WA 98195 USA; 3 Oregon Health and Science University, Knight Cancer Institute, Portland, OR 97239 USA; 4 University of Utah, Huntsman Cancer Institute, Salt Lake City, UT 84112 USA; 5 Ohio State University Comprehensive Cancer Center, Columbus, OH 43210 USA; 6 University of Colorado Anschutz Medical Campus, University of Colorado Cancer Center, Aurora, CO 80045 USA; 7 Wayne State University School of Medicine, Karmanos Cancer Institute, Detroit, MI 48201 USA; 8 University of Alabama Birmingham, O’Neal Comprehensive Cancer Center, Birmingham, AL 35233 USA; 9 University of Kansas Medical Center, Kansas City, KS 66103 USA; 10 University of Kentucky, Markey Cancer Center, Lexington, KY 40536 USA; 11 University of Indiana, Simon Cancer Center, Indianapolis, IN 46202 USA; 12 Washington University in St. Louis School of Medicine, Alvin J. Siteman Cancer Center, St. Louis, MO 63110 USA; 13 Vanderbilt-Ingram Cancer Center, Nashville, TN 37232 USA; 14 Ohio State University College of Medicine, Columbus, OH 43210 USA; 15 Department of Health Disparities Research, University of Texas, MD Anderson Cancer Center, Houston, TX 77030 USA; 16 University of Virginia Comprehensive Cancer Center, Charlottesville, VA 22903 USA

**Keywords:** COVID-19, Cancer, Health, Prevention, Behaviors

## Abstract

**Background:**

The COVID-19 pandemic involved business closures (e.g., gyms), social distancing policies, and prolonged stressful situations that may have impacted engagement in health behaviors. Our study assessed changes in cancer-related health behaviors during the pandemic, specifically physical activity, fruit/vegetable intake, smoking/tobacco use, and alcohol consumption.

**Methods:**

Eight cancer centers administered mailed/web-based/telephone surveys between June 2020 and March 2021. Surveys assessed demographics, perceptions on social distancing, and self-reported changes of behaviors (less/same/more) associated with cancer prevention or risk, e.g., physical activity, fruit/vegetable intake, tobacco/smoking use, and alcohol consumption. Descriptive analyses and logistic regression models assessed association of variables with behavior change.

**Results:**

Most of the 21,911 respondents reported adhering to at least 4(of 5) social distancing measures (72%) and indicated social distancing was very/somewhat important to prevent the spread of COVID-19 (91%). 35% of respondents reported less physical activity, 11% reported less fruit/vegetable intake, 27% reported more smoking/tobacco use (among those who used tobacco/smoking products in past 30 days), and 23% reported more alcohol consumption (among those who reported at least 1 drink in past 30 days) than before the pandemic. Urban residence, younger age, female gender, and worse general health were associated with less physical activity, less fruit/vegetable intake, more smoking/tobacco use, and more alcohol intake. Higher educational attainment was associated with less physical activity and fruit/vegetable intake and more alcohol consumption. Reporting social distancing as important and adhering to more COVID-19 safety practices were associated with less physical activity and more alcohol consumption.

**Conclusion:**

Our findings suggest that certain demographics and those who adhered to social distancing measures were more likely to self-report unfavorable changes in health behaviors during the pandemic. Future studies should examine if the behaviors returned to baseline following relief from pandemic restrictions, and if these behavior changes are associated with increased cancer incidence and mortality.

**Supplementary Information:**

The online version contains supplementary material available at 10.1186/s12885-024-13373-5.

## Background

The discovery and rapid spread of SARS-Cov2 in Wuhan, China in late 2019/early 2020 set off a worldwide COVID-19 pandemic that has resulted in millions of illnesses and deaths worldwide [1]. In response to the pandemic, the United States and countries across the globe instituted broad public health policies to reduce spread, including stay-at-home policies, limiting gatherings, social distancing and mask wearing indoors and, in some locations, outdoors [2, 3]. From the onset of the pandemic through vaccine distribution, these policies were implemented, followed, and enforced inconsistently across states in the US and even within different regions of individual states [3]. While implementation of these policies contributed substantially to the control of COVID-19, there has been concern regarding how COVID-19 impacted engagement in health behaviors that have shown to be associated with cancer risk, and specifically physical activity, fruit/vegetable intake, smoking/tobacco use, and alcohol consumption.

The alarms have already been sounded regarding potential detrimental consequences for cancer due to individuals delaying their cancer screenings, early therapy, and follow-up during the pandemic [4–6], yet changes to cancer risk related engagement in health behaviors present additional concern that may have a long-term impact on cancer incidence and mortality. A recent publication on changes in cancer risk-related health behaviors among cancer survivors reported that the most common reported pandemic-related change was engagement in physical activity, with a decrease in exercise reported for more than a third of participants [7]. Analyses of health behavior changes in large convenience samples of the general adult population early in the pandemic reported approximately one-third of respondents indicating an increase in tobacco use, alcohol consumption, and a decrease in physical activity [8–12]. In contrast, data from the 2020 National Health Interview Survey showed reduction in adult tobacco use during the first year of the COVID-19 pandemic, though it remained higher in rural than in urban communities [13]. 

Existing publications assessing COVID-19 and its association with cancer prevention behaviors are focused on specific populations or are framed in the context of cancer screening and cancer treatment [14–16]. Early publications describe the potential negative impact the pandemic can have on cancer patients’ psychosocial and physical wellbeing, and an even greater risk for negative behavior change among medically underserved communities, particularly related to nutrition. (14–15) Studies conducted via online survey reported that COVID-19 fear was associated with binge eating, decreases in physical activity, and increases in alcohol consumption. (16–17) Another study found decreases in physical activity, but no difference in diet quality [18]. To better understand responses to COVID-19 recommendations and identify groups that may be at higher risk for changes in cancer prevention behaviors, the National Cancer Institute (NCI) provided supplemental funding to multiple cancer centers located throughout the country to implement a common survey of cancer related health behaviors, cancer beliefs, and COVID-19 related behaviors. Our objective with this analysis was to assess changes in cancer-related behaviors after the onset of the COVID-19 pandemic among geographically diverse samples of U.S. adults. In this paper, we describe self-reported changes in cancer related engagement in health behaviors, including physical activity, fruit/vegetable consumption, smoking/tobacco use, and alcohol consumption from 21,911 participants across eight U.S. cancer centers.

## Methods

### Study population

Eight study sites provided data for this analysis, which included the University of Iowa, Ohio State University/Indiana University, Wayne State University/Karmanos Cancer Institute, University of Alabama at Birmingham, University of Colorado Anschutz Medical Center, Oregon Health and Science University, Vanderbilt University, and the University of Virginia. All sites administered questionnaires to individuals in their respective catchment areas between June 1, 2020 and March 31, 2021. Target populations, sampling frames, recruitment methods, mode, time frame and incentives differed across study sites and are described in Table 1. Centers used a variety of sampling and recruitment methods, which included convenience sampling, probability-based sampling, and recruitment from existing survey and cancer center databases (Table 1).

**Table 1:** Survey recruitment methods, mode, timeframe, and response rates by NCI-designated cancer center

University/ Cancer Center	Target Population	Sampling Frames	Modes	Timeframe	Incentives	Response Rate
University of Iowa - Holden Comprehensive Cancer Center	Survey 1: Iowa – general population Survey 2: Cancer center patients/ survivors	Survey 1: Probability-based sampling through Voter Registration File, oversampled rural populations Survey 2: Convenience sampling – recruited through cancer center research database (including ORIEN participants)	Survey 1: Mailed paper survey Survey 2: Online survey	Survey 1: Aug 11, 2020 – Dec 31, 2020 Survey 2: Sept 9, 2020 – Oct 20, 2020	Survey 1: $5 cash included with invitation Survey 2: Lottery for $50 gift card upon completion of survey	Survey 1: mailed 10,009 invitations, had 4,048 respondents (40%) Survey 2: emailed 2,954 invitations, had 780 respondents (26%)
Ohio State University Comprehensive Cancer Center	Ohio - general population and cancer center patients/ survivors; Indiana rural residents	Recruited prior participants from prior surveys targeting rural, Appalachian-area, minority, immigrant and LGBTQIA populations Convenience sampling – recruited through community partner connections and Pelotonia listserv	Online, mailed paper survey, or phone	June 19, 2020 – Dec 14, 2020	$10 gift card upon completion of survey	Sent 32,989 invitations, had 10,211 respondents (31%)
Wayne State University – Karmanos Cancer Institute	46 counties in Michigan including metro Detroit - general population and cancer patients/ survivors	Convenience sampling – recruited through 9 community-based organizations and via local news media and social media	Online	June 1, 2020 – Dec 31 2020	$10 gift card upon completion of survey	1,884 respondents
University of Alabama at Birmingham – O’Neal Comprehensive Cancer Center	12 counties in Alabama – general population	Convenience sampling – recruited through existing contacts via referrals from local community health advisors and at grocery stores and gas stations	Online or phone	Aug 14, 2020 – Mar 29, 2021	$25 upon completion of survey	799 participants were screened, and 616 completed a survey (77%)
University of Colorado Cancer Center	Colorado – general population	Recruited participants from the University of Colorado Health Survey Registry to draw a population-based sample – but oversampled rural, low-income, Latino and Black populations	Online, mailed paper survey, or phone	Aug 26, 2020 – Oct 17, 2020	$25 gift card upon completion of survey	1730 were invited and 1017 completed a survey (59%)
Oregon Health Science University – Knight Cancer Institute	Oregon – general population and cancer center patients/ survivors	Recruited prior survey participants from a population-based statewide Healthy Oregon Project cohort. Also used convenience sampling – recruited through healthcare and public health partners in rural areas of the state	Online and app-based	Aug 7, 2020 – Jan 12, 2021	$5 gift card upon completion of survey	Sent 7,990 population-based surveys, had 1,995 respondents (25%). Sent 7,655 invitations for convenience sample, had 1,154 respondents (15%)
Vanderbilt University – Ingram Cancer Center	All of Tennessee, 23 counties in western Kentucky and 5 counties in Alabama – general population	Recruited through database of cancer center stakeholders and community members/patients. Also used convenience sampling - recruited through ResearchMatch	Online	Dec 2, 2020 – Mar 18, 2021	$10 gift card upon completion of survey	1,084 respondents (no denominator)
University of Virginia Comprehensive Cancer Center	87 counties in Virginia and West Virginia – general population and cancer center patients/ survivors	Recruited prior participants of a catchment areas survey and ORIEN participants	Online or mailed paper survey	Nov 23, 2020 – Mar 1, 2021	$10 gift card upon completion of survey	Sent 5,777 invitations, had 1,682 respondents (29%)

### Measures

Investigators at the study sites collaborated to develop a core set of survey questions in addition to other questions that each site developed independently. The core set of survey questions was created using a combination of questions from existing surveys and newly developed Likert-type scale, multiple choice, and open-ended response items (see Appendix 1 for individual survey items and response options). Demographic survey items included age, gender, race, ethnicity, county of residence, health insurance coverage, marital status, household income, employment status, and education level. Health-related items included general health status and a summary score to assess comorbidities. Respondents’ county of residence was assigned as either rural or urban based on 2013 Rural-Urban Continuum Code (RUCC), with 1–3 categorized as urban and 4–9 as rural [19]. COVID-19 safety-related measures included perceived importance of social distancing as well as social distancing behaviors endorsed by study participants: staying at home except for going to work, outdoors to exercise, or going to the grocery store, pharmacy, or to get medical care; not having relatives, friends or neighbors come into your home; staying 6 feet away from people when you leave your home; wearing a face covering when you are inside a store or other place besides your home; and not attending public events. A variable measuring adherence to social distancing behaviors was created by combining all five social distancing behavior variables and used in analysis.

Engagement in health behaviors was measured including physical activity (“thinking about the past 30 days, in a typical week, how many days did you engage in any physical activity or exercise of at least moderate intensity?”), fruit/vegetable intake (“during the past 30 days, how often did you eat fruit?”, “during the past 30 days, how often did you eat vegetables other than potatoes?”), use of any smoking/tobacco products (“during the past 30 days, have you used any of the following tobacco and/or marijuana products?” with response options: cigarettes, little cigars, cigarillos, hand-rolled cigarettes, cigars, marijuana, pipe, bidi, smokeless tobacco or dip, electronic cigarettes containing nicotine, electronic cigarettes containing marijuana, hookah or waterpipe, other [please specify], and none), and alcohol consumption (“in the past 30 days, on how many days have you had a drink of an alcoholic beverage?”). Respondents were asked to report whether they changed the amount of engagement in each behavior compared to before the COVID-19 pandemic (more than before the pandemic, less than before the pandemic, same amount as before the pandemic). While these measures assessed perceived change in behaviors, we will refer to them as behavior changes throughout. In the models, reported changes were dichotomized as less vs. same/more for physical activity and fruit/vegetable intake whereas use of any smoking/tobacco products and alcohol consumption were dichotomized as more vs. same/less in our analyses.

### Coordination of data

UAB O’Neal Comprehensive Cancer Center served as the coordinating center for the study. UAB collected the de-identified survey response datasets from each participating site with IRB approval after conducting quality assessments of the data from each site. Potential data quality issues were discussed and resolved with investigators from each site before creating a homogenized dataset.

### Statistical analysis

Descriptive analyses included frequencies and proportions for categorical variables and means and standard deviations (SD) for continuous variables. Differences in the distributions of independent variables by behavioral changes were examined using Chi-square tests. Logistic regression was used to assess the bivariate associations of demographics, health status, importance of social distancing, and adherence to COVID-19 safety measures with each of the four self-reported behavior change variables (physical activity, fruit/vegetable intake, use of any smoking/tobacco products, and alcohol consumption). For each behavior variable, a multivariable-adjusted logistic regression model was developed to assess associations with: (1) less physical activity, (2) less fruit/vegetable intake, (3) more smoking/tobacco product use, and (4) more alcohol consumption, presented as odds ratios (ORs) with 95% confidence intervals (CIs). Model covariates included age, gender, race and ethnicity, rurality, health insurance coverage, marital status, household income, employment status, number of co-morbidities, importance of social distancing, and adherence to recommended COVID-19 social distancing measures. Data were analyzed using SAS 9.4 (SAS Institute, Cary, NC, USA). No weighting was used in analyses. P-values less than 0.05 were considered statistically significant.

## Results

Table 2 displays characteristics of the 21,911 survey respondents. Approximately 65% were 50 years or older, 83% identified as Non-Hispanic White, 68% were married, 55% were employed part- or full-time, 83% completed at least some college, and 65% lived in an urban county. Most participants rated their general health status as excellent, very good, or good (80%), with only 4% reporting their general health as poor. Almost three-quarters (72%) of participants reported that social distancing was ‘very important’ to preventing the spread of COVID-19, and 73% reported adhering to at least 4 out of 5 recommended COVID-19 safety measures.

**Table 2:** Characteristics of Survey respondents (*N* = 21,911)

Characteristic	*N*	%
Age (Years)		
Mean (Standard Deviation )	55.2 (16.1)	
Age-group		
18–34 years	2950	13%
35–49 years	4525	21%
50–64 years	6977	32%
65 + years	7186	33%
Missing	273	1%
Gender		
Male	6927	32%
Female	14,762	67%
Other (Transgender or Do not identify as male, female or transgender)	107	< 1%
Missing	115	1%
Race and Ethnicity		
Non-Hispanic White	18,197	83%
Non-Hispanic Black	1402	6%
Hispanic	950	4%
American Indian or Alaskan Native	95	< 1%
Asian or Asian American or Native Hawaiian or other Pacific Islander	418	2%
Arab	114	1%
Multi-Racial	173	1%
Missing	562	3%
Rurality		
Urban (RUCC^+^ 1–3)	14,272	65%
Rural (RUCC^+^ 4–9)	6717	31%
Missing	922	4%
Health Insurance Coverage		
No Insurance	933	4%
Public only	4595	21%
Private only	10,886	50%
Public and Private	4771	22%
Other or Unknown Insurance	424	2%
Missing	302	1%
Marital Status		
Single, never been married	2887	13%
Married/ Not married but living together	14,935	68%
Separated/ Divorced/ Widowed/ Other	3882	18%
Missing	207	1%
Combined Annual Income		
<$35,000	3761	17%
$35,000-$49,999	2240	10%
$50,000-$74,999	3693	17%
>=$75,000	9188	42%
Missing	3029	14%
Employment status		
Other	3005	14%
Retired	6504	30%
Employed Full-time/Part-time	12,059	55%
Missing	343	2%
Education		
High School or less	3568	16%
Some college/Associate degree	6823	31%
Bachelor’s degree	6186	28%
Master’s degree or higher	5173	24%
Missing	161	1%
General Health Status		
Excellent	2321	10%
Very good	7580	35%
Good	7714	35%
Fair	3364	15%
Poor	818	4%
Missing	114	1%
Number of co-morbidities		
Mean (Standard Deviation)	0.7 (0.9)	
Median (Range)	1 (0–6)	
Importance of social distancing		
Very important	15,760	72%
Somewhat important	4134	19%
A little important	1282	6%
Not important	551	3%
Missing	184	1%
Adherence to recommended COVID-19 Safety Measures		
0–2 safety measures	1763	8%
3 safety measures	4022	18%
4 safety measures	9958	45%
5 safety measures	6145	28%
Missing	23	< 1%
Physical activity frequency in last 30 days		
0 days per week	3396	16%
1–2 days per week	3242	15%
3 or more days per week	14,195	65%
Missing	1078	5%
Changes in Physical Activity During COVID-19 Pandemic		
Less	7633	35%
Same/More	13,799	63%
Missing	479	2%
Fruit OR Vegetable Consumption Score (from past 30 days) - Categories		
Q1: 0–1.14 servings/day	4505	21%
Q2: >1.14–2 servings/day	7289	33%
Q3: >2–3.43 servings/day	4105	19%
Q4: >3.43–20 servings/day	5173	24%
Missing	839	4%
Changes in Fruit/Vegetable Consumption During COVID-19 Pandemic		
Less	2503	11%
Same/More	18,848	86%
Missing	560	3%
Tobacco/ Marijuana Use in the past 30 days (Not mutually exclusive)		
Combustible tobacco use	2495	11%
Smokeless tobacco use	264	1%
E-cig use	453	2%
Marijuana use	1057	5%
Other tobacco/marijuana use	367	2%
Not used tobacco or marijuana	14,003	64%
Changes in Smoking/Tobacco Use During COVID-19 Pandemic^*^		
More	976	27%
Same/Less	2371	65%
Missing	323	9%
Alcohol Consumption in the past 30 days		
0 drinks (or missing)	9486	43%
1 or more drinks	12,425	57%
Binge Drinking in the past 30 days		
0 days of binge drinking in the past 30 days	18,227	83%
1 day of binge drinking in the past 30 days	828	4%
2 or more days of binge drinking in the past 30 days	1987	9%
Missing	869	4%
Changes in Alcohol Consumption During COVID-19 Pandemic^#^		
More	2827	23%
Same/Less	9426	76%
Missing	172	1%

+ Rurality was measured using 2013 Rural-Urban Continuum Codes (RUCC)

* Change in smoking was only among those who reported using smoking/tobacco products in the past 30 days, *n* = 3670

# Change in alcohol consumption was only among those who reported 1 or more drinks in the past 30 days, *n* = 12,425

Table 2 also shows that 65% of respondents reported three or more days of physical activity per week in the last 30 days, and 35% reported less physical activity during the COVID-19 pandemic (Table 2). 24% of respondents reported eating three or more servings of fruits and vegetables daily in the last 30 days, and 11% reported less fruit/vegetable intake during the COVID-19 pandemic. 64% of respondents reported no smoking/tobacco use in the last 30 days. Of those who reported any smoking/tobacco use in the last 30 days, 27% reported more smoking/tobacco use during the pandemic. The vast majority (83%) reported no episodes of binge drinking in the past 30 days. Among those who reported 1 or more drinks in the past 30 days, 23% reported more alcohol consumption during the pandemic.

Table 3 displays the unadjusted changes in physical activity and fruit/vegetable consumption during the pandemic reported by respondent characteristics. The youngest age group (aged 18–34 years) had a greater frequency of respondents that reported less physical activity during the pandemic (43% of 18–34 year-olds, 40% of 35–49, 33% of 50–64, and 33% of 65 or older, *p* < 0.001) and less fruit/vegetable intake during the pandemic (18% vs. 7% aged 65 and older, *p* < 0.001) compared to older age groups. Nearly half of respondents who reported poor general health also reported less physical activity during the pandemic, compared to a quarter of those who reported excellent general health (44% vs. 24%, *p* < 0.001). Those who reported poor general health also reported less fruit/vegetable intake during the pandemic more frequently than those in excellent health (19% vs. 7% excellent overall health, *p* < 0.001). Respondents who resided in urban areas more often reported less physical activity during the pandemic (39% urban vs. 28% rural, *p* < 0.0001). Less physical activity during the pandemic was more frequently reported by those who reported adherence to five COVID-19 safety measures (39% vs. 25% among those who adhered to 0–2 measures, (*p* < 0.001). Respondents of Hispanic ethnicity more often reported less fruit/vegetable intake during the pandemic (22% vs. 11% non-Hispanic White vs. 15% non-Hispanic Black, *p* < 0.001). Less fruit/vegetable intake during the pandemic was also reported among those with no health insurance (24% vs. 13% public insurance vs. 11% private insurance, *p* < 0.001).

**Table 3:** Changes in physical activity and Fruit/Vegetable intake during the COVID-19 pandemic

Variable	Changes in Physical Activity before and during the COVID-19 pandemic					Changes in Fruit and Vegetable before and during the COVID-19 pandemic				
	Less		Same/More			Less		Same/More		
	N	Row %	N	Row %	P Value^*^	N	Row %	N	Row %	P Value^*^
Age-group					< 0.001					< 0.001
18–34 years	1215	43%	1642	57%		501	18%	2337	82%	
35–49 years	1762	40%	2667	60%		723	17%	3665	83%	
50–64 years	2283	33%	4576	67%		773	11%	6054	89%	
65 + years	2285	33%	4750	67%		485	7%	6565	93%	
Gender					< 0.001					< 0.001
Male	2008	30%	4787	70%		602	9%	6168	91%	
Female	5551	38%	8898	62%		1865	13%	12,532	87%	
Other	52	50%	53	50%		25	25%	77	76%	
Race and Ethnicity					< 0.001					< 0.001
Non-Hispanic White	6135	34%	11,754	66%		1902	11%	15,916	89%	
Non-Hispanic Black	582	43%	772	57%		209	15%	1151	85%	
Hispanic	388	42%	529	58%		196	22%	715	78%	
Other	334	43%	437	57%		134	17%	637	83%	
Rurality					< 0.001					< 0.001
Urban	5465	39%	8504	61%		1738	13%	12,167	88%	
Rural	1828	28%	4733	72%		634	10%	5922	90%	
Health Insurance					< 0.001					< 0.001
No Insurance	358	40%	534	60%		218	24%	674	76%	
Public only	1541	34%	2944	66%		561	13%	3914	87%	
Private only	3771	35%	6963	65%		1200	11%	9467	89%	
Public and Private	1756	38%	2914	62%		438	9%	4230	91%	
Other + Unknown Insurance	128	32%	270	68%		45	11%	357	89%	
Marital Status					< 0.001					< 0.001
Single, never been married	1138	41%	1666	59%		450	16%	2325	84%	
Married/ Not married but living together	4981	34%	9687	66%		1524	10%	13,116	90%	
Separated/ Divorced/ Widowed/ Other	1450	38%	2338	62%		497	13%	3280	87%	
Combined Annual Income					< 0.001					< 0.001
<$35,000	1422	39%	2211	61%		639	18%	2975	82%	
$35,000-$49,999	837	38%	1354	62%		329	15%	1847	85%	
$50,000-$74,999	1351	37%	2265	63%		454	13%	3159	87%	
>=$75,000	3086	34%	6006	66%		841	9%	8219	91%	
Employment Status					< 0.001					< 0.001
Other	1239	43%	1666	57%		516	18%	2364	82%	
Retired	2100	33%	4267	67%		442	7%	5929	93%	
Employed Full-time/Part-time	4192	35%	7658	65%		1505	13%	10,288	87%	
Education					< 0.001					< 0.001
High School or less	994	28%	2446	71%		378	11%	3059	89%	
Some college/Associate degree	2515	38%	4148	62%		909	14%	5713	86%	
Bachelor’s degree	2186	36%	3903	64%		653	11%	5418	89%	
Master’s degree or higher	1896	37%	3215	63%		547	11%	4554	89%	
General health status					< 0.001					< 0.001
Excellent	546	24%	1743	76%		183	8%	2107	92%	
Very good	2331	32%	5137	69%		668	9%	6759	91%	
Good	2954	39%	4584	61%		939	13%	6578	87%	
Fair	1420	45%	1848	57%		555	17%	2711	83%	
Poor	351	44%	439	56%		144	19%	629	81%	
Number of co-morbidities					0.009					0.012
Mean (Standard Deviation)	0.7 (0.9)		0.7 (0.9)			0.7 (0.9)		0.7 (0.9)		
Median (Range)	1 (0–6)		0 (0–6)			0 (0–6)		1 (0–6)		
Importance of social distancing					< 0.001					0.001
Very important	5890	38%	9544	62%		1872	12%	13,519	88%	
Somewhat important	1236	31%	2802	69%		424	10%	3609	90%	
A little important	337	27%	924	74%		151	12%	1093	88%	
Not important	118	22%	419	78%		42	8%	490	92%	
Adherence to recommended COVID-19 Safety Measures					< 0.001					0.033
0–2 safety measures	425	25%	1283	75%		184	11%	1515	89%	
3 safety measures	1244	32%	2694	68%		415	11%	3511	89%	
4 safety measures	3632	37%	6099	63%		1170	12%	8533	88%	
5 safety measures	2329	39%	3717	61%		734	12%	5282	88%	

* P-values are assessing differences in covariates between those who had decreased vegetable consumption and those who had the same or more vegetable consumption. Chi-squared tests were used to compare variable distributions between groups

The unadjusted changes in tobacco/smoking use and alcohol consumption are shown in Table 4. More than a third of women who reported smoking/tobacco use in the past 30 days reported more use during the pandemic compared to only 20% of men (*p* < 0.001) (Table 4). Among urban respondents who reported smoking/tobacco use in the last 30 days, 33% reported more use during the pandemic than before the pandemic, vs. 22% of rural respondents (*p* < 0.001). Over 30% of smoking/tobacco users without health insurance reported more use since the start of the pandemic (*p* < 0.02). 33% of those who reported poor health also reported more tobacco use since the start of the pandemic, compared to only 28% of those who reported excellent overall health (*p* < 0.001). Over 30% of adults ages 18–34 and adults ages 35–49 that reported consuming alcohol in the past 30 days also reported more alcohol consumption since the start of the pandemic (*p* < 0.001) (Table 4). 26% of women, compared with only 17% of men, reported more alcohol consumption since the start of the pandemic (*p* < 0.001).

**Table 4:** Changes in Smoking/Tobacco Use and Alcohol Consumption during the COVID-19 pandemic

	Change to Tobacco Usage before and during the COVID-19 pandemic (*n* = 3670)					Change to Alcohol Consumption before and during the COVID-19 pandemic (*n* = 12425)				
Variable	More		Same/Less			More		Same/Less		
	N	Row %	N	Row %	P Value^*^	N	Row %	N	Row %	P Value^*^
Age-group										
18–34 years	295	36%	535	64%	< 0.001	575	32%	1205	68%	< 0.001
35–49 years	341	35%	638	65%		925	34%	1829	66%	
50–64 years	247	26%	712	74%		891	22%	3100	78%	
65 + years	86	16%	464	84%		408	11%	3183	89%	
Gender										
Male	262	20%	1048	80%	< 0.001	689	17%	3398	83%	< 0.001
Female	688	35%	1288	65%		2111	26%	5966	74%	
Other	22	47%	25	53%		19	35%	36	65%	
Race and Ethnicity										
Non-Hispanic White	734	28%	1909	72%	0.006	2446	23%	8097	77%	0.832
Non-Hispanic Black	77	32%	164	68%		148	23%	498	77%	
Hispanic	85	32%	152	64%		108	21%	396	79%	
Other	48	27%	82	63%		76	23%	256	77%	
Rurality										
Urban	702	33%	1449	67%	< 0.001	2130	26%	6148	74%	< 0.001
Rural	234	22%	823	78%		609	18%	2851	82%	
Health Insurance Coverage										
No Insurance	102	33%	206	67%	0.02	105	25%	324	75%	
Public only	285	28%	719	72%		339	17%	1670	83%	
Private only	465	31%	1053	69%		1993	28%	5102	72%	
Public and Private	101	24%	325	76%		327	14%	2097	86%	
Other + Unknown Insurance	12	21%	44	79%		46	22%	165	78%	
Marital Status										
Single, never been married	237	34%	460	66%	0.004	413	28%	1084	72%	< 0.001
Married/ Not married but living together	533	27%	1420	73%		2022	23%	6714	77%	
Separated/ Divorced/ Widowed/ Other	197	29%	472	71%		375	19%	1571	81%	
Combined Annual Income										
<$35,000	323	32%	691	68%	0.18	327	22%	1159	78%	< 0.001
$35,000-$49,999	120	27%	320	73%		239	21%	886	79%	
$50,000-$74,999	184	32%	396	68%		440	22%	1607	78%	
>=$75,000	298	29%	742	71%		1619	26%	4606	74%	
Employment Status										
Other	239	33%	491	67%	< 0.001	352	28%	891	72%	< 0.001
Retired	93	17%	667	83%		376	12%	2903	88%	
Employed Full-time/Part-time	627	31%	1390	69%		2076	27%	5538	73%	
Education										
High School or less	183	24%	576	76%	0.004	181	13%	1226	87%	< 0.001
Some college/Associate degree	413	30%	963	70%		740	21%	2773	79%	
Bachelor’s degree	244	31%	546	69%		1020	26%	2931	74%	
Master’s degree or higher	133	33%	273	67%		879	26%	2458	74%	
General health status										
Excellent	68	28%	178	72%	< 0.001	378	25%	1143	75%	0.281
Very good	245	26%	698	74%		1117	23%	3726	77%	
Good	345	28%	892	72%		933	22%	3213	78%	
Fair	238	35%	437	65%		340	24%	1108	76%	
Poor	76	33%	156	67%		52	20%	209	80%	
Number of co-morbidities										
Mean (Standard Deviation)	0.5 (0.8)		0.6 (0.9)		0.01	0.5 (0.7)		0.7 (0.8)		< 0.001
Median (Range)	0 (0–5)		0 (0–5)			0 (0–5)		0 (0–5)		
Importance of social distancing										
Very important	687	31%	1510	69%	0.004	2098	24%	6638	76%	0.002
Somewhat important	198	26%	551	74%		505	21%	1899	79%	
A little important	56	22%	194	78%		155	21%	573	79%	
Not important	32	26%	91	74%		60	19%	259	81%	
Adherence to recommended COVID-19 Safety Measures										
0–2 safety measures	71	19%	296	81%	< 0.001	215	21%	809	79%	< 0.001
3 safety measures	186	30%	445	71%		488	21%	1844	79%	
4 safety measures	443	30%	1052	70%		1191	23%	4030	77%	
5 safety measures	276	32%	577	68%		933	25%	2741	75%	

* P-values are assessing differences in covariates between those who had decreased vegetable consumption and those who had the same or more vegetable consumption. Chi-squared tests were used to compare variable distributions between groups

In adjusted regression analysis (Tables 5 and 6), younger adults (ages 18–34) had higher odds of reporting less physical activity during the pandemic (OR = 1.66, 95% CI: (1.42, 1.95)), less fruit/vegetable intake during the pandemic (OR = 2.70, 95% CI: (2.12, 3.44)), more smoking/tobacco use during the pandemic (OR = 2.96, 95% CI: (1.84, 4.76)), and more alcohol consumption during the pandemic (OR = 2.52, 95% CI: (1.95, 3.25)), in comparison to the oldest age group (ages 65 or older). Compared to female gender, male gender was associated with lower odds of having less physical activity (male OR = 0.73, 95% CI: (0.68, 0.78)), less fruit/vegetable consumption (male OR = 0.78, 95% CI: (0.69, 0.86)), more smoking/tobacco use (male OR = 0.54, 95% CI: (0.44, 0.66)), and more alcohol consumption during the pandemic (male OR = 0.71, 95% CI: 0.64, 0.79)). Compared to non-Hispanic White respondents, Hispanic respondents had greater odds of reporting less fruit/vegetable intake during the pandemic (OR = 1.27, 95% CI: (1.04, 1.56)). Rural residence was associated with lower odds of having less physical activity (rural OR = 0.72, 95% CI: (0.67, 0.78)), less fruit/vegetable intake (rural OR = 0.88, 95% CI: (0.79, 0.99)), and more alcohol consumption during the pandemic (rural OR = 0.74, 95% CI: (0.64, 0.84)) compared to urban respondents.

**Table 5:** Univariate and Multivariable-adjusted logistic regression models for less physical activity and Fruit/Vegetable intake

	Less Physical Activity		Less Fruit/Vegetable Intake	
	Unadjusted Model	Adjusted Model	Unadjusted Model	Adjusted Model
Age Group				
18–34 years	1.54 (1.41–1.68)*	1.66 (1.42–1.95)*	2.90 (2.54–3.32)*	2.70 (2.12–3.44)*
35–49 years	1.37 (1.27–1.49)*	1.41 (1.22–1.64)*	2.67 (2.36–3.02)*	2.39 (1.91–2.99)*
50–64 years	1.04 (0.97–1.11)	1.22 (1.07–1.39)*	1.73 (1.54–1.95)*	1.93 (1.57–2.37)*
65 + years	REF	REF	REF	REF
Gender				
Female	REF	REF	REF	REF
Male	0.67 (0.63–0.72)*	0.73 (0.68–0.78)*	0.66 (0.59–0.72)*	0.78 (0.69–0.86)*
Other	1.57 (1.07–2.31)*	1.11 (0.73–1.69)	2.18 (1.39–3.43)*	1.22 (0.74–2.04)
Race and Ethnicity				
Non-Hispanic White	REF	REF	REF	REF
Non-Hispanic Black	1.44 (1.29–1.62)*	1.11 (0.97–1.27)	1.52 (1.30–1.77)*	1.18 (0.99–1.40)
Hispanic	1.41 (1.23–1.61)*	1.13 (0.97–1.32)	2.29 (1.94–2.71)*	1.27 (1.04–1.56)*
Other	1.46 (1.27–1.69)*	1.20 (1.01–1.42)*	1.76 (1.45–2.13)*	1.25 (1.00–1.55)*
Rurality				
Urban	REF	REF	REF	REF
Rural	0.60 (0.56–0.64) *	0.72 (0.67–0.78)*	0.75 (0.68–0.83)*	0.88 (0.79–0.99)*
Health Insurance Coverage				
No Insurance	REF	REF	REF	REF
Public only	0.78 (0.67–0.91)*	0.85 (0.71–1.02)	0.44 (0.37–0.53)*	0.71 (0.57–0.89)*
Private only	0.81 (0.70–0.93)*	0.83 (0.70–0.99)*	0.39 (0.33–0.46)*	0.60 (0.48–0.74)*
Public and Private	0.90 (0.78–1.04)	1.08 (0.89–1.32)	0.32 (0.27–0.38)*	0.91 (0.71–1.17)
Other + Unknown Insurance	0.71 (0.55–0.91)*	0.74 (0.54–1.00)*	0.39 (0.28–0.55)*	0.61 (0.40–0.93)*
Marital Status				
Single, never been married	REF	REF	REF	REF
Married/ Not married but living together	0.75 (0.69–0.82)*	1.00 (0.90–1.12)	0.60 (0.54–0.67)*	1.00 (0.86–1.15)
Separated/ Divorced/ Widowed/ Other	0.91 (0.82–1.00)	1.10 (0.98–1.25)	0.78 (0.68–0.89)*	1.15 (0.97–1.36)
Combined Annual Income				
<$35,000	REF	REF	REF	REF
$35,000 - $49,999	0.96 (0.86–1.07)	1.04 (0.92–1.18)	0.83 (0.72–0.96)*	1.00 (0.84–1.18)
$50,000 - $74,999	0.93 (0.84–1.02)	1.06 (0.94–1.19)	0.67 (0.59–0.76)*	0.87 (0.74–1.02)
>=$75,000	0.80 (0.74–0.87)*	0.94 (0.84–1.06)	0.48 (0.43–0.53)*	0.65 (0.55–0.77)*
Employment Status				
Other	REF	REF	REF	REF
Retired	0.66 (0.61–0.72)*	0.90 (0.79–1.04)	0.34 (0.30–0.39)*	0.65 (0.53–0.80)*
Employed Full-time/Part-time	0.74 (0.68–0.80)*	0.89 (0.80–0.99)*	0.67 (0.60–0.75)*	0.93 (0.81–1.06)
Education				
High School or less	REF	REF	REF	REF
Some college/Associate degree	1.49 (1.37–1.63)*	1.46 (1.31–1.63)*	1.29 (1.13–1.46)*	1.47 (1.25–1.72)*
Bachelor’s degree	1.38 (1.26–1.51)*	1.46 (1.30–1.64)*	0.98 (0.85–1.25)	1.34 (1.13–1.60)*
Master’s degree or higher	1.45 (1.32–1.59)*	1.56 (1.38–1.76)*	0.97 (0.85–1.12)	1.55 (1.29–1.87)*
General health status				
Excellent	0.49 (0.44–0.54)*	0.43 (0.38–0.49)*	0.61 (0.52–0.72)*	0.52 (0.43–0.64)*
Very good	0.70 (0.66–0.75)*	0.67 (0.62–0.72)*	0.69 (0.62–0.77)*	0.70 (0.62–0.79)*
Good	REF	REF	REF	REF
Fair	1.19 (1.10–1.30)*	1.13 (1.03–1.25)*	1.43 (1.28–1.61)*	1.38 (1.21–1.58)*
Poor	1.24 (1.07–1.44)*	1.16 (0.98–1.39)	1.60 (1.32–1.95)*	1.31 (1.04–1.67)*
Number of co-morbidities	1.04 (1.01–1.08)*	0.98 (0.94–1.03)	0.94 (0.90–0.99)*	0.99 (0.93–1.05)
Importance of social distancing				
Very important	2.19 (1.78–2.70)*	1.48 (1.14–1.93)*	1.62 (1.17–2.22)*	1.45 (0.98–2.13)
Somewhat important	1.57 (1.26–1.94)*	1.25 (0.96–1.63)	1.37 (0.99–1.91)	1.27 (0.86–1.87)
A little important	1.30 (1.02–1.65)*	1.18 (0.89–1.57)	1.61 (1.13–2.31)*	1.43 (0.94–2.16)
Not important	REF	REF	REF	REF
Adherence to recommended COVID-19 Safety Measures				
0–2 safety measures	REF	REF	REF	REF
3 safety measures	1.39 (1.23–1.59)*	1.15 (0.98–1.35)	0.97 (0.81–1.17)	0.90 (0.72–1.14)
4 safety measures	1.80 (1.60–2.02)*	1.27 (1.09–1.48)*	1.13 (0.96–1.33)	0.96 (0.77–1.20)
5 safety measures	1.89 (1.68–2.13)*	1.29 (1.10–1.51)*	1.14 (0.96–1.36)	1.02 (0.81–1.28)

Models adjusted for age, gender, race and ethnicity, rurality, health insurance coverage, marital status, household income, employment status, number of co-morbidities, importance of social distancing, and adherence to recommended COVID-19 social distancing measures

Statistical significance at *p* < 0.05 is noted by an asterisk (*)

**Table 6:** Univariate and Multivariable-adjusted logistic regression models for more Tobacco usage and alcohol consumption

	More Tobacco Usage (*n* = 3670)		More Alcohol Consumption (*n* = 12425)	
	Unadjusted Model	Adjusted Model	Unadjusted Model	Adjusted Model
Age Group				
18–34 years	2.98 (2.27–3.90)*	2.96 (1.84–4.76)*	3.72 (3.23–4.30)*	2.52 (1.95–3.25)*
35–49 years	2.88 (2.21–3.76)*	2.46 (1.56–3.88)*	3.95 (3.47–4.49)*	2.59 (2.04–3.29)*
50–64 years	1.87 (1.43–2.46)*	1.86 (1.22–2.86)*	2.42 (1.97–2.55)*	1.68 (1.34–2.11)*
65 + years	REF	REF	REF	REF
Gender				
Female	REF	REF	REF	REF
Male	0.47 (0.40–0.55)*	0.54 (0.44–0.66)*	0.57 (0.52–0.63)*	0.71 (0.64–0.79)*
Other	1.65 (0.92–2.94)	1.02 (0.55–1.91)	1.49 (0.85–2.61)	0.95 (0.52–1.73)
Race and Ethnicity				
Non-Hispanic White	REF	REF	REF	REF
Non-Hispanic Black	1.22 (0.92–1.62)	0.92 (0.67–1.28)	0.98 (0.81–1.19)	0.82 (0.66–1.02)
Hispanic	1.45 (1.10–1.92)*	1.01 (0.72–1.40)	0.90 (0.73–1.12)	0.65 (0.51–0.83)*
Other	1.52 (1.06–2.20)*	1.27 (0.84–1.92)	0.98 (0.76–1.28)	0.68 (0.51–0.90)*
Rurality				
Urban	REF	REF	REF	REF
Rural	0.59 (0.50–0.70) *	0.78 (0.64–0.96)*	0.62 (0.56–0.68)*	0.74 (0.64–0.84)*
Health Insurance				
No Insurance	REF	REF	REF	REF
Public only	0.80 (0.61–1.05)	0.82 (0.57–1.17)	0.63 (0.49–0.80)*	0.82 (0.61–1.09)
Private only	0.89 (0.69–1.16)	0.92 (0.66–1.29)	1.21 (0.96–1.51)	0.91 (0.70–1.18)
Public and Private	0.63 (0.45–0.87)*	1.03 (0.67–1.59)	0.48 (0.38–0.62)*	0.85 (0.62–1.17)
Other + Unknown Insurance	0.55 (0.28–1.09)	0.48 (0.20–1.12)	0.86 (0.58–1.28)	0.85 (0.54–1.33)
Marital Status				
Single, never been married	REF	REF	REF	REF
Married/ Not married but living together	0.73 (0.61–0.88)*	0.87 (0.69–1.10)	0.79 (0.70–0.90)*	1.14 (0.98–1.34)
Separated/ Divorced/ Widowed/ Other	0.81 (0.65–1.02)	1.04 (0.79–1.38)	0.63 (0.53–0.74)*	1.02 (0.85–1.24)
Combined Annual Income				
<$35,000	REF	REF	REF	REF
$35,000 - $49,999	0.80 (0.63–1.03)	0.95 (0.71–1.26)	0.96 (0.79–1.15)	0.93 (0.75–1.15)
$50,000 - $74,999	0.99 (0.80–1.24)	1.21 (0.92–1.59)	0.97 (0.83–1.14)	0.94 (0.77–1.14)
>=$75,000	0.86 (0.71–1.04)	1.08 (0.82–1.43)	1.25 (1.09–1.43)*	1.00 (0.83–1.20)
Employment Status				
Other	REF	REF	REF	REF
Retired	0.43 (0.33–0.57)*	0.92 (0.61–1.40)	0.33 (0.28–0.39)*	0.67 (0.53–0.85)*
Employed Full-time/Part-time	0.93 (0.77–1.11)	1.01 (0.80–1.27)	0.95 (0.83–1.08)	0.96 (0.82–1.13)
Education				
High School or less	REF	REF	REF	REF
Some college/Associate degree	1.35 (1.10–1.65)*	1.25 (0.98–1.60)	1.81 (1.52–2.16)*	1.52 (1.24–1.87)*
Bachelor’s degree	1.41 (1.12–1.76)*	1.33 (1.01–1.76)*	2.36 (1.99–2.80)*	1.82 (1.48–2.23)*
Master’s degree or higher	1.53 (1.18–2.00)*	1.53 (1.10–2.12)*	2.42 (2.04–2.88)*	2.02 (1.63–2.49)*
General health status				
Excellent	0.99 (0.73–1.34)	0.78 (0.53–1.13)	1.13 (0.99–1.31)	0.98 (0.84–1.15)
Very good	0.91 (0.75–1.10)	0.97 (0.78–1.21)	1.03 (0.94–1.14)	1.02 (0.91–1.14)
Good	REF	REF	REF	REF
Fair	1.41 (1.15–1.72)*	1.55 (1.23–1.96)*	1.06 (0.92–1.22)	1.19 (1.01–1.41)*
Poor	1.26 (0.93–1.70)	1.45 (1.00–2.09)	0.86 (0.63–1.17)	1.12 (0.78–1.60)
Number of co-morbidities	0.90 (0.82–0.98)*	1.00 (0.89–1.13)	0.74 (0.70–0.78)*	0.90 (0.84–0.97)*
Importance of social distancing				
Very important	1.29 (0.86–1.96)	0.79 (0.47–1.34)	1.36 (1.03–1.81)*	1.57 (1.10–2.25)*
Somewhat important	1.02 (0.66–1.58)	0.70 (0.41–1.18)	1.15 (0.85–1.55)	1.27 (0.89–1.82)
A little important	0.82 (0.50–1.36)	0.69 (0.39–1.24)	1.17 (0.84–1.63)	1.23 (0.84–1.81)
Not important	REF	REF	REF	REF
Adherence to recommended COVID-19 Safety Measures				
0–2 safety measures	REF	REF	REF	REF
3 safety measures	1.74 (1.28–2.38)*	1.43 (1.05–2.23)*	1.00 (0.83–1.19)	0.86 (0.69–1.08)
4 safety measures	1.76 (1.32–2.33)*	1.30 (1.02–2.08)*	1.11 (0.94–1.31)	0.88 (0.71–1.09)
5 safety measures	1.99 (1.48–2.68)*	1.33 (1.04–2.18)*	1.28 (1.08–1.52)*	0.94 (0.76–1.17)

Models adjusted for age, gender, race and ethnicity, rurality, health insurance coverage, marital status, household income, employment status, number of co-morbidities, importance of social distancing, and adherence to recommended COVID-19 social distancing measures

Statistical significance at *p* < 0.05 is noted by an asterisk (*)

Perceptions of importance and adherence to social distancing were associated with greater odds of behavioral changes for physical activity and alcohol consumption. Those who reported that social distancing was very important in preventing the spread of COVID-19 also had significantly greater odds of reporting less physical activity (OR = 1.48, 95% CI: (1.14, 1.93)) (Table 5) and more alcohol consumption during the pandemic (OR = 1.57, 95% CI: (1.10, 2.25)) (Table 6). Those who reported adherence to five COVID-19 safety measures had greater odds of reporting less physical activity during the pandemic (OR = 1.29, 95% CI: 1.10, 1.51) compared to those who adhered to 0–2 safety measures (Table 3).

## Discussion

In this survey of populations in study sites across the U.S., a substantial percentage of respondents reported less physical activity and fruit/vegetable intake, and more smoking/tobacco use and alcohol consumption during the pandemic vs. pre-pandemic, which were generally associated with living in urban areas, younger age, female gender, worse general health, higher educational attainment, and perceived importance of, and adherence to, social distancing measures. Historically, many of these factors, including urban residence, younger age, female gender, and higher educational attainment, have been associated with more favorable cancer-related prevention behaviors [20–24]. Our results suggest that the pandemic was associated with unique patterns of engagement in negative behavior change in these populations. While we were not able to specially evaluate the mechanisms by which this may have occurred, we hypothesize that these changes could be related to issues accessing healthy foods and areas to safely exercise, or could reflect the additional mental health strain caused by the COVID-19 pandemic. Increases in stress, anxiety, and depression may discourage people from participating in healthy cancer prevention behaviors. (25–26) Those who were non-adherent to social distancing measures presumably did not substantially alter their behaviors during the pandemic, whereas those who were more adherent likely had to make significant changes to their typical daily routines and experienced higher stress levels leading to more negative behaviors.

Prioritization of COVID-19 mitigation behaviors in response to the immediate risk of infection were critical in controlling the pandemic, though they may have unintended consequences for longer-term health. Nearly three-quarters of respondents reported that that they believed social distancing was very important and that they adhered to at least four COVID-19 safety measures; these individuals tended to have greater odds of negative behavior change. While social distancing measures and business closures aimed to keep individuals safe from COVID-19, it appears they may have resulted in more behavior changes associated with cancer and other chronic diseases. Most respondents reported high adherence of COVID-19 mitigation behaviors, but in prioritizing mitigation and their immediate health (not getting/spreading COVID-19), cancer-related behaviors may have taken a backseat. Consequently, we now need to address gaps that were created in cancer and other chronic disease prevention behaviors which may compromise longer-term health if left unaddressed.

While structural barriers, such as business closures, likely contributed to behavior changes in our population, there is also evidence to suggest that the stress and isolation resulting from the pandemic worsened mental health [27–29]. Stress, isolation, and worsened mental health may have led to negative behavior changes reported by respondents. Our findings that adherence to more COVID-19 safety measures was associated with less physical activity and perceiving social distancing as important was associated with more alcohol consumption during the pandemic are consistent with this rationale.

Our findings are also largely consistent with other studies related to the COVID-19 pandemic which have reported that younger age, female gender, residing in an urban area, and higher educational attainment were associated with greater odds for decreased physical activity [8–10]. Women frequently assumed more family caretaking responsibilities during the pandemic with school and work closures [30–32]. This additional stress may have led to less time to focus on one’s behavioral health. A larger proportion of our study population reported less physical activity (34%) compared to a convenience sample population that was surveyed via social media (12%).^8^

Our findings highlight the importance of being better prepared for the next pandemic or other situations requiring social distancing/isolation by leveraging lessons learned during the COVID-19 pandemic. Reviews, including Bentlage et al., focused on identifying behavior changes among specific populations, as well as gathering practical recommendations for maintaining an active lifestyle during the COVID-19 pandemic [33–36]. Development of educational resources on evidence-based strategies for maintaining healthy behaviors during isolation situations will better prepare populations and providers to mitigate negative health effects of these types of events. This approach would likely have broad health benefits beyond cancer prevention, as these behaviors are associated with a wide spectrum of common chronic illnesses, such as diabetes and heart disease [37]. 

These findings are also important to consider as post-COVID-19 data continue to emerge in relation to cancer incidence, treatment, and mortality. The pandemic limited access to cancer screenings and follow-up that is necessary for timely diagnosis and favorable cancer-related outcomes. The pandemic also limited access to environments that foster healthy cancer-related prevention behaviors, such as exercise facilities and stores that provide access to fresh fruits and vegetables. The combined impact of limited cancer-related medical care and increases in negative behaviors could result in increased incidence rates as well as a higher frequency of advanced-stage cancers that require more complex treatment. It is important for clinicians to prioritize counseling patients about prevention behaviors, encouraging screening and facilitating timely diagnosis to avoid an increase in the burden of cancer and other chronic diseases in their patient populations.

The main strength of this study is its implementation in the first year of the pandemic, when interruptions to behavior were likely at their greatest. Also, surveys were developed and implemented by large study teams within cancer centers and academic institutions with expertise in survey methodology. As we know that not all individuals were impacted by the COVID-19 pandemic in the same way, the diversity of the sample across age, general health status, geographic location, and race/ethnicity provided insight into the many experiences during the first year of the pandemic. This study also builds on the limited body of literature investigating the association between the COVID-19 pandemic and engagement in cancer-related risk behaviors and assesses the association of demographic characteristics and COVID-19-related concerns with behavior change. This body of literature is important for future research as we continue to track cancer incidence/mortality and build understanding of cancer-related behaviors to improve response to future widespread events that impact health. Finally, the items in the survey were created using items from previous surveys, allowing comparison across samples in different contexts.

This study is not without limitations. The survey was cross-sectional in nature and therefore we are unable to establish causality and temporality of demographic characteristics and health behavior change. Recall bias could impact the accuracy of responses related to behaviors that occurred prior to the pandemic. Questions and responses were framed as changes from pre-pandemic to help minimize errors in recalling specific details of behaviors prior to the pandemic. Another major limitation was that the sampling frame of surveys included convenience sampling in some areas, limiting generalizability of results to the U.S. population. In addition, due to the large number of study sites and needing to balance feasibility with length of the survey, some items were omitted that might have provided additional context for the associations of included survey items, including cancer survivor status. This study assessed perceived (i.e., self-reported) behavior change rather than objective measures of behavior change. It is possible that systematic errors such as social desirability bias could have inflated reporting of desirable behaviors. However, it would seem that this would affect all behavior change and perceived importance variables in the same direction serving to attenuate our main finding.

## Conclusions

Our findings regarding changes in cancer risk behaviors present concern over how the pandemic could impact cancer incidence and mortality in the coming years. Should the observed changes in behaviors be sustained, we may see increased cancer incidence in the future, though this may not be apparent for decades due to the long period of development of some cancers. Due to this, it is important to understand whether these behaviors have been sustained since relief from COVID-19 restrictions and to closely monitor cancers associated with physical activity and tobacco use. (38–39) A sustained decrease in physical activity could result in an increased risk for, and incidence, of bladder, breast, colon, endometrial, esophageal, kidney, and stomach cancers [38]. A similar pattern could be observed with a sustained increase in tobacco/smoking use, with an increased risk and incidence of oral cavity, esophageal, lung, liver, stomach, kidney, pancreatic, uterine, bladder, and colorectal cancers [39]. Additionally, the public health impact of these increased risk behaviors could extend beyond cancer risk. For example, decreased physical activity and fruit/vegetable intake and increased alcohol consumption can be associated with high blood pressure and cholesterol, stroke, type 2 diabetes, and chronic heart disease [40]; increased smoking/tobacco usage can be associated with chronic lung disease, stroke, and heart disease thus setting the stage for inducing or exacerbating comorbid conditions [41]. 

It is necessary for researchers and clinicians to consider steps that can be taken to identify and provide additional resources/services for those whose cancer risk behaviors were most impacted by the COVID-19 pandemic. Furthermore, our results highlight other important public health outcomes and the potential for unintended consequences of public health safety measures. Public health leaders, healthcare providers and policymakers should consider exploring strategies to support and enhance healthy behaviors during and after public health emergencies to allow for better balance in the future between minimizing risk of contracting the illness and promoting behaviors that optimize physical and mental health.

## Electronic supplementary material

Below is the link to the electronic supplementary material.

Supplementary Material 1

## Data Availability

Raw survey data are not publicly available due to data privacy laws. Reasonable requests for aggregated data can be submitted to the coordinating center at the University of Alabama at Birmingham.

## Abbreviations

COVID-19: Coronavirus disease (SARS-CoV-2 virus)
NCI: National Cancer Institute
RUCC: Rural-Urban Continuum Code
IRB: Institutional Review Board
UAB: University of Alabama at Birmingham
SD: Standard deviation
OR: Odds ratio
CI: Confidence interval
