# Supplementary material for: Cancer-related health behaviors during the COVID 19 pandemic in geographically diverse samples across the US
Source: BMC Cancer. 2025 Jan 9;25:50. doi: 10.1186/s12885-024-13373-5 (PMC11721185; doi:10.1186/s12885-024-13373-5)
Supplement: Supplementary file 1 — Supplementary Material 1 [file 12885_2024_13373_MOESM1_ESM.docx]

**Appendix 1**. Source of core items, sorted by survey item, with response options and question type included.

| **#** | **Survey Item** | **Response Options** | **Type** | **Source** |
| --- | --- | --- | --- | --- |
| 1 | Have you been tested for COVID-19 by a medical doctor or health care professional? | Yes, I was tested, and it showed I had/have COVID-19; Yes, I was tested, and it showed I did NOT have COVID-19; Yes, I was tested and am waiting for results; No, I tried to get tested but could not get a test; No, I have not tried to get tested; Don’t know/not sure | Covariate | Stanford |
| 2 | In the past 30 days, have you been in close physical contact with a person who has tested positive for coronavirus or COVID-19? | Yes; No; Don’t know, not sure | Covariate | Stanford |
| 3 | Since [DATE], what types of social distancing are you doing most or all of the time?  3a: Staying at home except for going to work, outdoors to exercise, or going to the grocery store, pharmacy, or to get medical care?  3b: Not having relatives, friends, or neighbors come into your home?  3c: Staying 6 feet away from people when you leave your home?  3d: Wearing a face covering when you are outdoors?  3e: Wearing a face covering when you are inside a store or other place besides your home? | Yes; No; Don’t know, not sure | Social Distancing | Ohio Surveillance Survey |
| 4 | Since [DATE], when COVID-19 restrictions began, have you attended the following?  4a: Any gatherings, not including work, with more than 2 people who do not live in the same house as you?  4b: A rally or demonstration of 20 or more people?  4c: Other large social gatherings of 20 people or more? | Yes; No; Don’t know, not sure |  | Ohio Surveillance Survey |
| 5 | How important do you think social distancing is during COVID-19? | Very important; somewhat important; A little important; Not important; Don’t know, not sure | Social Distancing | Adapted from SHOW |
| 6 | In the past 2 weeks, how often have you received support from friends or loved ones to help you during the COVID-19 pandemic? | Every day; Several times a week; Once a week; Once in 2 weeks; Never; Don’t know, not sure | Social Distancing | N2-COVID-19 Check-In |
| 7 | In general, would you say that your health is? | Excellent; Very good; Good; Fair; Poor; Don’t know, not sure | Covariate | BRFSS |
| 8 | Has a doctor ever diagnosed you with any of the following conditions? | Heart disease; lung disease; ulcer or stomach disease; liver disease; cancer; osteoarthritis or degenerative arthritis; Rheumatoid arthritis; high blood pressure; diabetes; kidney disease; anemia or other blood disease; depression; back pain; HIV; other (specify) | Covariate | Adapted from BRFSS |
| 9 | Has your clinic, doctor’s office or dental practice closed or cancelled an appointment because of COVID-19? | Yes; No; Did not need an appointment; Don’t know, not sure | COVID Constraints | Adapted from Kalichman COVID-19 Assessment |
| 10 | Have you cancelled a clinic, doctor or dental appointment to avoid being around others? | Yes; No; Did not need an appointment; Don’t know, not sure | COVID Constraints | Adapted from Kalichman COVID-19 Assessment |
| 11 | Have you been unable to obtain one or more prescription medication because of the COVID-19 pandemic? | Yes; No; Did not need to obtain prescription medication(s); Don’t know, not sure | COVID Constraints | Adapted from Kalichman COVID-19 Assessment |
| 12 | Have you been unable to obtain one or more over-the-counter medicines you needed because of the COVID-19 pandemic? | Yes; No; Did not need to obtain over-the-counter medication(s); Don’t know, not sure | COVID Constraints | Adapted from Kalichman COVID-19 Assessment |
| 13 | Thinking about the last 30 days, in a typical week, how many days did you do any physical activity or exercise of at least moderate intensity, such as brisk walking, bicycling at a regular pace, and swimming at a regular pace? | ___ Days | DV | CITIES Study |
| 14 | Have you changed the frequency of your physical activity compared to BEFORE the COVID-19 pandemic? | Yes, I have engaged in MORE; Yes, I have engaged in LESS; No, I have been doing the SAME; Don’t know, not sure | COVID Perception | CITIES Study |
| 15 | During the past 30 days, not including juices, how often did you eat fruit? [include fresh, frozen or canned fruit. Do not include dry fruits]. You may specify the number of times per day, per week, or per month, whichever is easiest for you. Write the number of times in the box below, and then check if it is the number of times per day, per week, or per month | __ Number of times  _Per day _Per week _Per month | DV | BRFSS |
| 16 | During the past 30 days, how often did you eat vegetables other than potatoes? Include things like a salad, cooked dried beans, corn, and broccoli. You may specify the number of times per day, per week, or per month, whichever is easiest for you. Write the number of times in the box below, and then check if it is the number of times per day, per week, or per month. | __ Number of times  _Per day _Per week _Per month | DV | BRFSS |
| 17 | Have you changed the amount of fruit and vegetables you consume per day compared to BEFORE the COVID-19 pandemic? | Yes, I have consumed MORE; Yes, I have consumed LESS; No, I have been consuming the SAME; Don’t know, not sure | COVID Perception | BRFSS |
| 18 | In the past 30 days, on how many days have you had a drink of an alcoholic beverage? | __ Days | DV | N2 COVID-19 Check-In |
| 19 | In the past 30 days, on how many days did you have 5 or more alcoholic beverages on the same occasion? | __ Days |  | N2 COVID-19 Check-In |
| 20 | Have you changed the amount of alcohol you drink compared to BEFORE the COVID-19 pandemic? | Yes, I have drank MORE; Yes, I have drank LESS; No, I have drank the SAME; Don’t know, not sure | COVID Perception | N2 COVID-19 Check-In |
| 21 | During the past 30 days, have you used any of the following tobacco and/or marijuana products? | Cigarettes; little cigars; cigarillos; hand-rolled cigarettes; cigars (without marijuana); blunts (with marijuana); marijuana (rolled in a paper); pipe; bidi; smokeless tobacco or dip; electronic cigarettes containing nicotine; electronic cigarettes containing marijuana; hookah or waterpipe; other products (please specify); I have not used any tobacco products in the past 30 days | DV | New question developed for this survey |
| 22 | Have you changed the frequency of tobacco and/or marijuana use compared to BEFORE the COVID-19 pandemic? | Yes, I have used MORE; Yes, I have used LESS; No, I have been using the SAME; Don’t know, not sure | COVID Perception | N2 COVID-19 Check-In |
| 23 | Are you currently on oral medication for your cancer? | Yes; No; Don’t know, not sure | DV | New question developed for this survey |
| 24 | In the past week, about how many days did you take your oral cancer medication? | __ Days | DV | New question developed for this survey |
| 25 | Has your oral cancer medication use changed during the COVID-19 pandemic? | Yes, I used it MORE often; Yes, I used it LESS often; No, I have used it the SAME amount | COVID Perceptions | New question developed for this survey |
| 26 | Were you scheduled for any cancer-related medical care that you had to cancel or reschedule during the COVID-19 restrictions? | Yes; No; Don’t know, not sure | COVID Constraints | New question developed for this survey |
| 27 | What did you have to cancel or reschedule? Check all that apply. | Routine appointment; screening test; blood test; surgery; chemotherapy; radiation therapy; therapy (physical or occupational); other (please specify) | DV | New question developed for this survey |
| 28 | What is your ZIP code? |  |  | BRFSS |
| 29 | County |  |  | BRFSS |
| 30 | What is your date of birth? |  |  |  |
| 31 | What is your race? | White; Black or African American; American Indian or Alaska Native; Asian or Asian American; Native Hawaiian or Pacific Islander; Arab; Other (please specify) |  | RISE Study |
| 32 | Are you of Hispanic origin? | Yes; No |  | BRFSS |
| 33 | What sex were you assigned at birth, on your original birth certificate? | Male; Female; Prefer not to answer |  | Williams Institute |
| 34 | How do you describe yourself? | Male; Female; Transgender; Do not identify as female, male, or transgender; Prefer not to answer |  | CITIES LBTQ |
| 35 | Do you consider yourself to be… | Heterosexual or straight; Gay or lesbian; Bisexual; Other (please specify); Prefer not to answer |  | BRFSS |
| 36 | What is the highest grade or level of school you completed? | Less than high school; Some high school, no diploma; GED/High school graduate; Some college but no degree; Associate Degree – occupational/vocational; Associate degree – academic program; Bachelor’s degree; Master’s degree; Professional school degree; Doctorate degree |  | CERTS Project 3 |
| 37 | Which category best describes your occupational status in February 2020 prior to the stay-at-home orders put in place as a result of the COVID-19 pandemic? | Employed full-time; Employed part-time; Unemployed; Homemaker; Student; Retired; Disabled; Other (please specify) |  | New question developed for this survey |
| 38 | Are you currently being paid for a full-time or part-time job, including being paid by a job while you stay at home? Do not include unemployment compensation. | Yes; No |  | Ohio Surveillance Survey |
| 39 | Since March 1^st^, did you lose a job because of COVID-19? | Yes; No |  | Ohio Surveillance Survey |
| 40 | Thinking about members of your family living in your household, what is the combined annual income, meaning the total pre-tax income from all sources earned in the past year? | $0-$9,999; $10,000-$14,999; $15,000-$19,999; $20,000-$34,999; $25,000-$49,999; $50,000-$74,999; $75,000-$99,999; $100,000-$199,999; $200,000 or more |  | HINTS C5 Cycle 1: O19 |
| 41 | How would you describe your household’s financial situation right now? | After paying the bills, you still have enough money for special things you want; You have enough money to pay the bills, but little extra money to buy something that you want; You have money to pay the bills, but only because you have cut back on things you want; You are having difficult paying the bills no matter what you do |  | RISE Study |
| 42 | What is your current marital status? | Single, never been married; Married; Not married but living together; Separated; Divorced; Widowed; Other (please specify) |  | CERTS Project 3 |
| 44 | Are you covered by health insurance or some other kind of health care plan? | Yes / No |  | RISE Study |
| 45 | What type of health insurance do you have? Select all that apply. | Medicaid; Private health insurance; Medicare; Medicare plus a supplemental policy; Military/VA; Other (please specify) |  | RISE Study |
| 46 | How many adults live in your household including you? |  |  | CARE II |
| 47 | How many children (less than 18 years of age) live in your household? | __ Please provide the age and gender of each child. |  | SHOW |
